# Polyelectrolytes for Environmental, Agricultural, and Medical Applications

**DOI:** 10.3390/polym16101434

**Published:** 2024-05-18

**Authors:** Martina Zuñiga Delgado, Francisca L. Aranda, Fabian Hernandez-Tenorio, Karla A. Garrido-Miranda, Manuel F. Meléndrez, Daniel A. Palacio

**Affiliations:** 1 Departamento de Polímeros, Facultad de Ciencias Químicas, Universidad de Concepción, Edmundo Larenas 129, Casilla 160-C, Concepción 4070409, Chilefaranda@udec.cl (F.L.A.); 2 Department of Materials Engineering (DIMAT), Faculty of Engineering, University of Concepcion, 270 Edmundo Larenas, Box 160-C, Concepcion 4070409, Chile; 3 Environmental Processes Research Group, School of Applied Sciences and Engineering, Universidad EAFIT, Medellin 050022, Colombia; fehernandt@eafit.edu.co; 4 Scientific and Technological Bioresource Nucleus (BIOREN-UFRO), Universidad de La Frontera, Temuco 4780000, Chile; karla.garrido@ufrontera.cl; 5 Facultad de Ciencias para el Cuidado de la Salud, Universidad San Sebastián, Campus Las Tres Pascuales, Lientur 1457, Concepción 4060000, Chile

**Keywords:** polyelectrolyte, environmental, medicine, agriculture

## Abstract

In recent decades, polyelectrolytes (PELs) have attracted significant interest owing to a surge in research dedicated to the development of new technologies and applications at the biological level. Polyelectrolytes are macromolecules of which a substantial portion of the constituent units contains ionizable or ionic groups. These macromolecules demonstrate varied behaviors across different pH ranges, ionic strengths, and concentrations, making them fascinating subjects within the scientific community. The aim of this review is to present a comprehensive survey of the progress in the application studies of polyelectrolytes and their derivatives in various fields that are vital for the advancement, conservation, and technological progress of the planet, including agriculture, environmental science, and medicine. Through this bibliographic review, we seek to highlight the significance of these materials and their extensive range of applications in modern times.

## 1. Introduction

**Figure 1:**
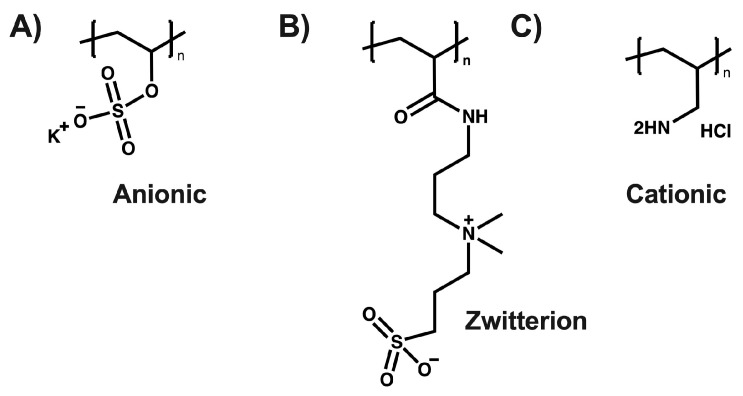
(**A**) poly(vinyl sulfate) potassium, (**B**) poly[(3(methacryloylamino)propyl)dimethyl(3-sulfopropyl)ammonium hydroxide], and (**C**) poly(allylamine hydrochloride).

In recent decades, polyelectrolytes have remained a highly relevant research topic due to their significant utility in the development of technologies and biological applications. Polyelectrolytes are compounds that exhibit net positive or negative charges depending on pH, with their solubility dictated by the interactions among these charges [1,2] (See Figure 1). These macromolecules, due to their ionizable nature, can dissociate in polar solvents such as water, resulting in counterions dispersed throughout the solution with opposite charges along the chain. Generally, most macromolecules with these characteristics are soluble in water, endowing them with unique properties stemming from the electrostatic interactions present [3,4,5]. 

Due to the electrostatic interactions between these macromolecules and the microdomains of polyelectrolyte components, a variety of applications emerge. Generally, in environmental research, they have been utilized for the removal of contaminants such as antibiotics, metal ions, dyes, and other concerning pollutants [6,7]. Moreover, in agriculture, they serve as strategies for soil remediation or as fertilizers [8,9]. Particularly in the medical field, they play a crucial role in the development of new approaches in life sciences, necessitating new tools to address the challenges in less invasive, more cost-effective medical practices with high efficiency. These include drug carriers targeting specific organs to enhance drug availability or reduce toxicity [10,11,12,13]. This review article aims to provide a general overview of the polyelectrolyte applications in environmental research, agriculture, and medicine, and how they have contributed to the development of technologies and materials to enhance the overall quality of life on the planet.

## 2. Bibliometric Analysis

The unique properties of polyelectrolytes, such as ionic strength, viscosity, pH sensitivity, ionization constant, and solubility, among others, render these macromolecules a current topic of interest in the scientific community. Therefore, it was pertinent to analyze the trends regarding the utilization of polyelectrolytes in applications such as medicine, agriculture, and the environment. Consequently, a systematic search was conducted in the Scopus scientific database using the criteria established in the following equation: TITLE-ABS-KEY (“polyelectrolytes” AND (“medicine” OR “agriculture” OR “environmental”)) AND (LIMIT-TO (DOCTYPE, “ar”) OR LIMIT-TO (DOCTYPE, “re”)). The scientometric tools utilized included the Bibiometrix software (University of Naples Federico II, Naples, Italy) within R Commander (×64. 4.1.0), VOSviewer version 1.6.16 (Leiden University, the Netherlands), and CorText Manager (INRAE, Noisy-le-Grand, France). These tools were employed to calculate parameters such as the total number of citations, average citations per article, and the categorization of highly cited publications. Additionally, bibliometric networks were constructed, including co-authorship and co-occurrence maps, contingency matrices, Sankey diagrams, and historical maps. It is noteworthy that the collected documents were filtered to avoid term repetition with abbreviations and hyphens [14].

### 2.1. Scientific Production

**Figure 2:**
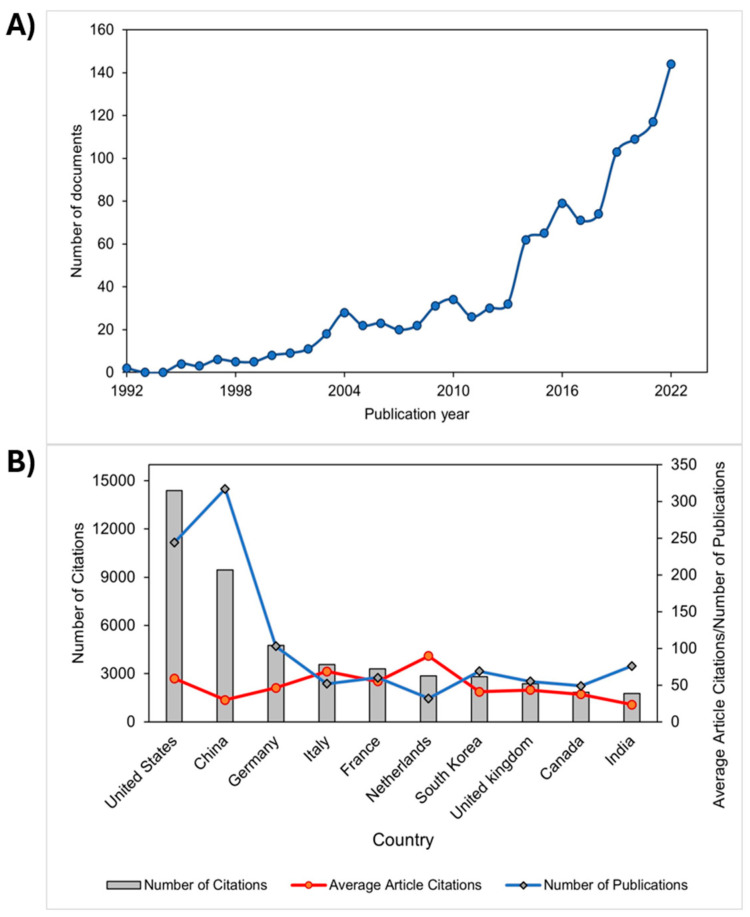
(**A**) Trend of increasing publications on polyelectrolytes and (**B**) leading countries in polyelectrolyte publications and their applications, based on Scopus reports.

The scientific publication output demonstrated an increasing trend in research on polyelectrolytes in the fields of agriculture, medicine, and the environment. Figure 2A showed a significant increase of 79.17% over the last decade from 2012 to 2022. This trend is likely related to the growing interest from industrial and academic environments in fundamental and applied research on polyelectrolytes. Studies worldwide are intensifying due to the immense potential of polyelectrolytes in advanced technology in areas such as agriculture, medicine, environmental remediation, and materials science [15]. Additionally, the increasing trend also showed that, by the year 2021, 56.89 % of the total number of publications had more than 10 citations, while by the year 2022, the number of publications with more than 10 citations was 38.77 %. These results indicate that polyelectrolyte studies are relevant in the field of scientific research and are used as important references for other research.

On another note, countries with the highest scientific production in polyelectrolyte studies were categorized (See Figure 2B). It was found that the United States showed the highest contribution in citations (14,384), followed by China (9450) and Germany (4763). Additionally, these countries exhibited the highest number of publications with 244, 317, and 103 documents, respectively. Therefore, the leadership in research on polyelectrolytes in medical, environmental, and agricultural applications by these three countries is notable. Moreover, the positive impact generated by these publications in the field of study and their likely use as important references for other innovative developments can be inferred. It is worth noting that the categorization was conducted based on the nationalities of the corresponding authors.

The bibliometric analysis also allowed the identification of the most-cited publications on polyelectrolytes and their applications. Table 1 shows the most impactful document with 2167 citations and 164.53 citations per year, corresponding to a review article published by authors from research centers in the United States and Italy [16]. This review described new developments in bioapplications using chitosan, which was analyzed as an interesting polyelectrolyte for medical applications due to its non-toxicity to living tissues, reactive functional groups, high adsorption capacity, and gel-forming ability. Additionally, the researchers highlighted the development of the networks of polyelectrolyte complexes based on chitosan with DNA; proteins such as albumin, gelatin, collagen, keratin, and fibroin; anionic polysaccharides such as chondroitin sulfate, glucosaminoglycans, heparin, hyaluronic acid, pectin, alginate, xanthan gum, carboxymethyl cellulose, and dextran sulfate; and anionic polymers such as polyacrylic acid. Similarly, an important research article was identified for its contributions to the topic with 603 citations and 83.71 citations per year.

### 2.2. Co-Authorship Analysis and Co-Occurrence

**Figure 3:**
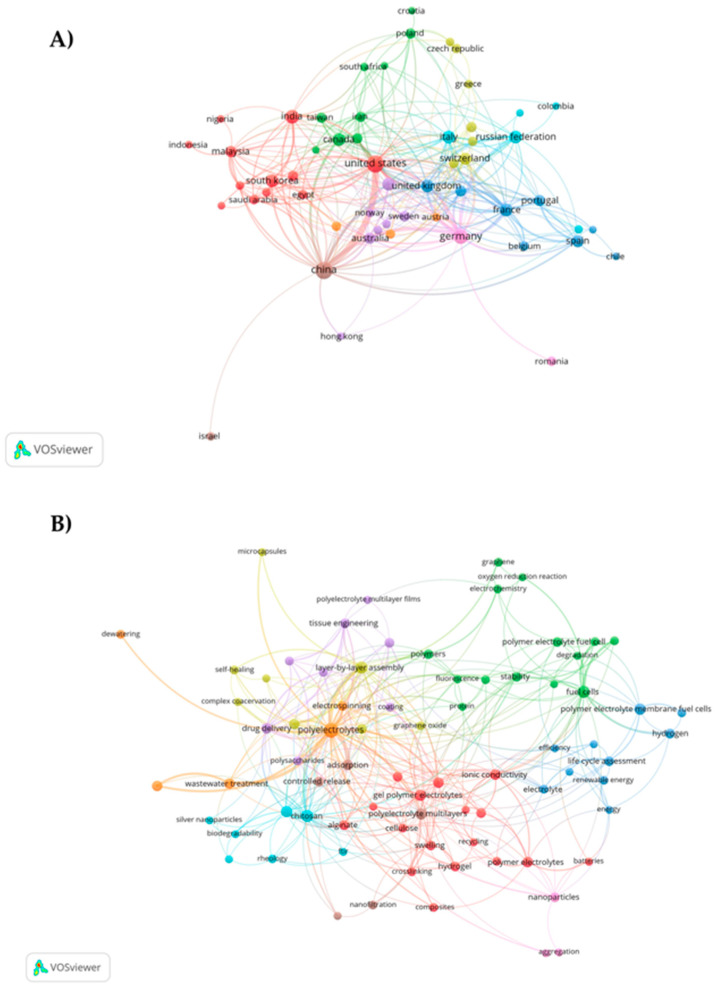
(**A**) Map of co-authorship of countries in publications on polyelectrolytes and their applications. Note: seven groups of countries: red, green, light blue, purple, yellow, blue, and dark red and (**B**) co-occurrence map of author keywords in publications on polyelectrolytes and their applications. Note: seven thematic groups: yellow, blue, green, purple, red, orange, and light blue.

The co-authorship and co-occurrence analyses were conducted with the purpose of investigating a broad spectrum of issues such as scholarly communication, research front, and the intellectual structure of research fields and to study cross-disciplines related to the topic of polyelectrolytes [25]. Additionally, Liu and Mei [26] claimed that the use of these analyses enhances the ability of quantitative techniques to represent the structural and dynamic aspects of scientific research. They also make it possible to identify and link the specialized literature not only within a given period but also over time. Figure 3A depicts the collaboration network among countries in studies on polyelectrolytes and their applications. It was found that the United States exhibited the highest cooperation in research, with a network consisting of 11 countries. Additionally, it was identified that this network is interconnected with the one led by China. It is noteworthy that the United States and China were the leading countries in scientific production on the topic. The results presented demonstrate the relationship between the researchers and their respective institutional affiliations, highlighting scientific cooperation in the production of novel materials based on polyelectrolytes capable of generating new developments and applications in the fields of medicine, agriculture, and the environment.

On the other hand, the keyword co-occurrence map revealed seven interconnected topic clusters (See Figure 3B). These grouped themes were related to applications such as wastewater treatment, tissue engineering, battery development, drug delivery, renewable energy, and fuel cells, among others. Similarly, natural polyelectrolytes like chitosan, alginate, cellulose, and proteins were identified. Additionally, the map provided significant information regarding the characterization and properties of polyelectrolytes (stability, rheology, efficiency, aggregation, ionic conductivity, FT-IR, biodegradability, adsorption, and fluorescence, among others). Moreover, the trends in formulation methods such as hydrogels, layer-by-layer assembly, nanoparticles, and microencapsulation were determined. On the other hand, the main keywords were determined based on their co-occurrence, where polyelectrolytes (150), layer-by-layer assembly (47), chitosan (46), full cells (43), and polyelectrolyte complexes (36) were the words most closely associated in the published research according to the data extracted from Scopus.

### 2.3. Contingency Matrix, Sankey Diagram, and Historical Map

**Figure 4:**
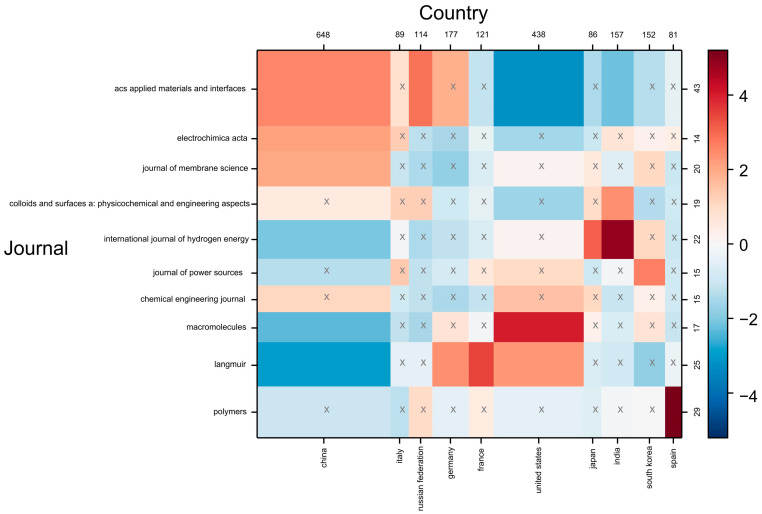
Contingency matrix on the relationship between journals and countries in polyelectrolyte publications and their applications (cells with x indicate that the model deviation is not statistically significant).

The contingency matrix involved an analysis that allowed the identification of the correlations between the bibliometric parameters. The matrix was developed to visualize the relationship and magnitude of co-occurrence between the leading countries in the polyelectrolyte scientific publications and specialized journals. The colors indicated the degree of correlation between two variables under the measure of a statistical co-occurrence metric Chi-square. On the numerical scale, a value of four indicated that the observed co-occurrence was 400% higher than the expected value (See Figure 4). Conversely, a value of −4 showed that the observed co-occurrence result was 400% lower than expected. The matrix presented white cells indicating that the variables had no relationship (neutrality). Additionally, negative correlations were observed through blue cells, while red cells signified a strong relationship [27]. Figure 4 displays the correlations between the most relevant journals and countries in the scientific production of polyelectrolytes and their applications. The matrix indicated that the United States had a correlation of four with the journal “Macromolecules” (Q1), meaning there were four times more articles assigned between these factors than would be expected if the co-occurrence distributions and semantic groups were independent [28]. Also, a correlation of three was observed between China and the journal “ACS Applied Materials & Interfaces” (Q1). It is noteworthy that “Macromolecules” and “ACS Applied Materials & Interfaces” are journals of the American Chemical Society, indicating the high quality of publications produced by these countries.

**Figure 5:**
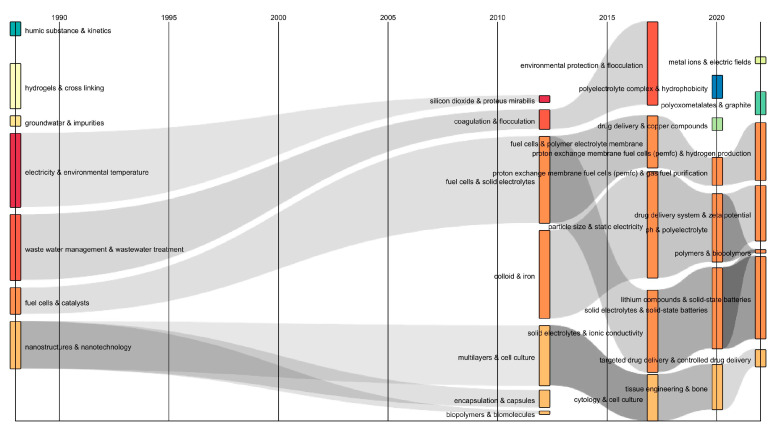
Sankey diagram of author keywords in polyelectrolyte publications and their applications.

Also, a Sankey diagram was constructed to represent the evolution and connection of the different topics related to polyelectrolytes over a time scale in an efficient manner. The diagram showed interrelated keywords through the flows of gray color [29], with the combinations of keywords “full cells & catalysts” and “nanostructures & nanotechnology” being prominent during the period of 1990–2010 (See Figure 5). These combinations converged into various combinations such as “full cells & solid electrolytes”, “solid electrolytes & ionic conductivity”, “solid electrolytes & solid-state batteries”, and “lithium compounds & solid-state batteries”, and “multilayers & cell culture”, “cytology & cell culture”, “tissue engineering & bone”, and “targeted drug delivery & controlled drug delivery” during the periods 2010–2015, 2015–2020, and 2020–2022, respectively. Hence, it can be inferred from the temporal flows that polyelectrolytes have been highly relevant in the generation of energy devices, tissue engineering, and the design of systems for targeted drug delivery, owing to their unique physical properties such as adjustable surface charge, the absence of glass transitions, insolubility in common organic solvents, stable structures, and high surface hydrophilicity, making them ideal candidates for the development of proton exchange membranes in fuel cells [30]. Additionally, polyelectrolytes meet the profile requirements of biocompatible polymer systems and can be adapted as carriers and components for active substances [1]. It is noteworthy that the divergence and convergence of streams, as well as the transformations of keywords, showed the dynamic evolution of the polyelectrolyte research field over time.

**Figure 6:**
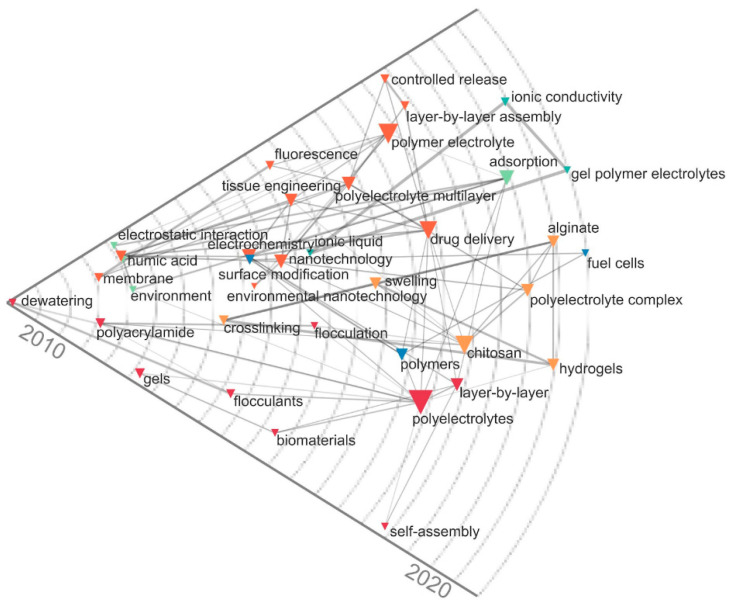
Historical map of keyword co-occurrence in polyelectrolyte publications and their applications.

Additionally, the historical map was developed in the period 2010–2020 and showed the relationships between the most important keywords according to the co-occurrence measures of the scientific publications on polyelectrolytes, (See Figure 6). This analysis complemented the flows observed in the Sankey diagram, highlighting developments in tissue engineering, fuel cells, wastewater treatment (flocculation systems), and drug delivery in the last decade. Additionally, a trend towards the use of gel polymer electrolytes in fuel cell production was observed, as this type of material can be used as liquid solutions providing electrochemical properties, solid hosts ensuring mechanical strength, and added fillers enhancing ionic conductivity [31]. In general, the bibliometric analysis was important to establish the current trends on polyelectrolytes and the dynamics of scientific research from the linkage of the specialized literature. Also, the analysis makes a significant contribution to the study of technological advances in polyelectrolytes and provides some representative suggestions to researchers working on the subject.

## 3. Application

Polyelectrolytes have garnered significant attention in the scientific community due to their diverse range of applications, ranging from drug delivery, the removal of emerging contaminants such as antibiotics and metal ions, soil substrates, and dialysis membranes, to contact lenses, and more [8,32,33,34]. Here, we will explore the applications of polyelectrolytes focused on the environmental, agricultural, and medical domains.

It is important to mention that among the applications of polyelectrolytes, events such as the conformation of polyelectrolytes in solution play a fundamental role in their diverse applications. The interaction between the opposite charges of the functional groups in the polymer and the ions in the surrounding medium influences their three-dimensional structure. For example, in environments with a high concentration of ions, polyelectrolytes can adopt more extended configurations due to electrostatic repulsion [35,36].

Additionally, the conformation of polyelectrolytes has a significant impact on the capture of metals in solution. Under specific pH and ionic strength conditions, polyelectrolytes can form three-dimensional complexes with metal ions through electrostatic and coordination interactions [37]. The structure and flexibility of the polymer influence its metal-capturing capacity, as they determine the accessibility of the charged functional groups to interact with the metal ions [38]. Furthermore, changes in the polymer’s conformation in response to environmental conditions can affect the stability and efficacy of the metal–polyelectrolyte complex. These interactions between conformation and metal capture provide a deeper understanding of how polyelectrolytes can be applied in different contexts and environments [39].

### 3.1. Environmental

One of the major global concerns is water scarcity and environmental pollution caused by organic and inorganic pollutants. In recent years, research in this field has intensified, with polyelectrolytes playing a significant role in the development of polymeric materials aimed at reducing or eliminating these contaminants. Polyelectrolytes, owing to their functionality, possess groups capable of interacting with such pollutants. While standalone polyelectrolytes may exhibit limitations such as low reuse cycles and removal capacities, combinations with various inorganic materials like clays, zeolites, SiO_2_, TiO_2_, and organic materials like cellulose and polystyrene have addressed these drawbacks [40]. A wide range of pollutants has become a global concern, including dyes, pesticides, herbicides, drugs, and heavy metals, among others [41,42,43].

#### 3.1.1. Organic Pollutants

In recent years, the global presence of organic contaminants, particularly pharmaceuticals, has raised significant concerns regarding their impact on aquatic ecosystems. This issue has spurred extensive research across multiple disciplines due to the emergence of antibiotic-resistant bacterial strains, associated public health risks, and challenges in medical treatment efficacy caused by the presence of pharmaceuticals in various environmental matrices. For instance, Palacio et al. investigated the removal of antibiotics from aqueous solutions using quaternary ammonium-modified chitosan coupled with ultrafiltration membranes. Their study achieved removal percentages exceeding 80% and the maximum retention capacities of 185.5, 420.2, and 632.8 mg g^−1^ for ciprofloxacin, amoxicillin, and tetracycline, respectively [44]. Similarly, Quinlan et al. explored the use of quaternized chitosan beads for the removal of 2-naphthoxyacetic acid from aqueous solutions, achieving a 91% removal rate and a maximum retention capacity of 315 mg g^−1^ [45]. Fei et al. employed alginate beads with graphene oxide for the removal of ciprofloxacin from aqueous systems, demonstrating a maximum retention capacity of 86.12 mg g^−1^ [46]. Moreover, Palacio et al. investigated the efficacy of three copolymers with different charge ratios in removing tetracycline and ciprofloxacin from aqueous solutions, with all three copolymers achieving removal rates exceeding 70% [33,47]. Kasgoz et al. utilized polyelectrolyte nanocomposite hydrogels with clays and 3-acrylamide-2-methylpropanesulfonic acid sodium salt for the removal of dyes such as Safranine-T (ST) and Brilliant Cresyl Blue (BCB) in aqueous solutions, achieving maximum retention capacities of 484.2 and 494.2 mg g^−1^ for ST and BCB, respectively [48]. Mahdavinia et al. obtained nanocomposite hydrogels based on carrageenan and laponite for the removal of cationic dyes such as crystal violet in aqueous solutions, with results showing a 79.8 mg g^−1^ retention capacity [49], and Panic et al. obtained hydrogels based on poly(methacrylic acid) for the absorption of cationic dyes such as basic yellow 28, with removal percentages exceeding 70% and a maximum retention capacity of 157 mg g^−1^ [50]. Wang et al. removed methylene blue using nanocomposite hydrogels of alginate and smectite clay, demonstrating rapid adsorption capacity and a retention capacity of 1843.5 mg g^−1^, presenting this material as an absorbent with high retention capacity compared to other materials reported in the literature [51]. El-Zahhar et al. removed bromophenol blue from industrial waters by synthesizing polymer–clay composites from poly(acrylamide co-acrylic acid) and kaolinite, achieving removal of the contents of 10.78 mg g^−1^ [52]. Bhattacharyya et al. utilized nanocomposites of polyacrylic acid and polyethylene glycol with nano clays for the removal of Congo Red and methyl violet in water, with removal studies showing maximum retention capacities of 2227 and 1927 mg g^−1^ for Congo Red and Methyl Violet, respectively [53].

In other studies, graphene nanosheets [54] and nanolaminate–alginate hydrogels via ionic gelation have been used for the elimination of a mixture of emerging contaminants, including bisphenol A, ofloxacin, and diclofenac [55]. Additionally, materials supported on alginate-lime (Fe-Cu/Alg-LS) have been fabricated for the removal of medications such as ciprofloxacin (CIP) and levofloxacin (LEV), proving effective in contaminant removal [56]. Membrane technologies, particularly bionic three-layer SA/GO/CS membranes using sodium alginate (SA), chitosan (CS), and graphene (GO) [57], have achieved the elimination of 99.987% of contaminants, presenting superior separation performance for water treatment across a wide spectrum [58].

#### 3.1.2. Inorganics Pollutants

**Figure 7:**
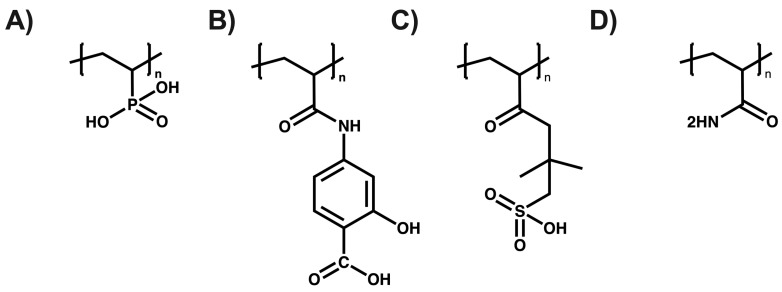
(**A**) Poly (vinylphosponic acid), (**B**) Poly(N-methacryloyl-4-aminosalicylic acid), (**C**) Poly(2-acrylamido-2-methyl-1-propanesulfonic acid), and (**D**) Poly(acrylamide).

Urbano et al. have reported the utilization of polyelectrolytes in the removal of inorganic contaminants in the form of ion exchange resins made from poly(sodium 4-styrene sulfonate) and poly(2-acrylamido glycolic acid) for the removal of metal ions such as Cd(II), Pb(II), Cu(II), Cr(III), and Al(III), achieving retention percentages above 80% after 1 h of contact [59]. Rivas et al. utilized poly(diallyldimethylammonium chloride) coupled with ultrafiltration membranes for the removal of molybdenum (VI) and vanadium (V), obtaining maximum retention capacities of 370 mg g^−1^ for molybdenum (VI) and 520 mg g^−1^ for vanadium (V) [60]. Polowczyk et al. employed resins containing quaternary ammonium groups and N-methyl-D-glucamine to remove chromium in aqueous solutions, achieving a maximum retention capacity of 677.9 mg g^−1^ [61]. Perez et al. developed interpenetrating hybrid absorbents using cationic polymers such as poly(4-(vinylbenzyl)trimethylammonium chloride) and zirconium oxides for arsenic removal, where absorbents with a higher hydrous zirconium oxide content showed a greater sorption of arsenite, and those with the lowest content exhibited a greater sorption of arsenate [62]. Mohamed et al. produced crosslinked hydrogels with glutaraldehyde from quaternized chitosan and poly(vinyl alcohol), applying them in the removal of metal ions such as Ni^2+^, Co^2+^, Cu^2+^, Cr^3+^, Fe^3+^, and Cd^2+^, achieving removal percentages above 70% for all the metals studied [63,64]. Furthermore, with the purpose of illustrating the structural diversity of polyelectrolytes and highlighting the wide range of options available for metal removal, the structures of the various polyelectrolytes used in the removal of the metal ions are presented. These are provided as representative examples to visually complement the general discussion on the design and functionality of polyelectrolytes in purification applications (see Figure 7) [65,66].

In other studies, the utilization of chitosan-based hydrogels for the removal of copper (II) from wastewater has been reported, and when modifying the hydrogel with zirconium, removal increases between 15 and 50 wt% [67]. Some authors have prepared chitosan–zirconium beads by encapsulation for the removal of boron (III) in wastewater [68]. Meanwhile, other authors have modified chitosan with zirconium for the removal of phosphates in agricultural wastewater [69]. The use of chitosan in the form of beads, crosslinked with GLA (glutaraldehyde), has enabled the removal of bovine serum albumin, showing favorable adsorption at pH values ranging from 4.7 to 6.0. Crosslinking methods have also been employed for the removal of nitrates and orthophosphates with a mixture of P-PO_4_, N-NO_2_, and N-NO_3_, investigating the effect of the crosslinking agent on the removal of different nutrients. The interaction mediated by the number of hydrogen bonds between H_2_PO_4_- and NH_2_ along with -OH is stronger than that between N-NO_2_ and N-NO_3_, resulting in a higher removal of orthophosphates than nitrates [70].

#### 3.1.3. Applications in Contaminated Soils

Another major concern regarding environmental issues is the decreasing habitability of soil due to rapid industrialization and overpopulation [71]. This problem varies greatly depending on urbanization, competitive surroundings, and anthropogenic emissions in general [72,73]. Consequently, soil contaminants leach into rivers, causing water pollution through deforestation, heavy metals [74], pesticides, biological pathogens, and the different types of micro/nanoplastic particles [75,76,77]. All the aforementioned factors have resulted in physicochemical changes in the soil. For instance, more than 60% of cardiovascular diseases are related to soil pollution, and 70% of non-communicable diseases are also affected by it [78,79]. 

Among the primary interests in soil improvement is soil stabilization, which refers to altering or preserving one or more soil properties to enhance engineering characteristics [80]. Bio-stabilization involves the use of biological products or processes [81]. Biopolymers, consisting of biology-based additives, can be used as agents to improve soil permeability. Various biomaterials such as xanthan gum, carrageenan, and chitosan have been employed for soil stabilization [82,83].

Furthermore, a wide variety of materials allow for the bioremediation of contaminated systems. One technique involves the use of enzymes and/or microorganisms [84]. Researchers have found that the degradation of soil contaminants mediated by bacteria requires absorbing contaminants through molecular mobility, which implies a slow process [85,86]. Therefore, enzymes are included in calcium alginate microspheres to enhance the resistance and capacity of polyelectrolyte materials, offering practical applications in contaminated sites [85]. In recent years, research has focused on bioremediation and nanobioremediation mediated by polyelectrolytes derived from natural sources. For instance, alginate compounds with polyethyleneimine (PEI) clay and PEI-montmorillonite have been prepared and encapsulated in alginate beads for the adsorption and biodegradation of formaldehyde in water [87]. Another technique involves nanobioremediation with various types of nanoparticles to achieve more cost-effective and environmentally friendly processes to mitigate different types of contaminants [88]. 

Additionally, the use of alginate–chitosan polyelectrolyte complexes incorporating montmorillonite to enhance encapsulation efficiency and interaction properties with contaminants has been explored [89]. Inorganic materials such as halloysite and graphene have been utilized in networks of polyelectrolyte complexes [90]. Furthermore, studies have reported the use of humic acid for soil remediation [91]. Other studies have utilized sodium alginate, chitosan, and activated carbon composite materials for the sustained release of humic acid to provide soil remediation and increase agricultural production with the addition of nitrogen, phosphorus, potassium, and organic matter [91,92,93]. Significant progress has been made in natural polyelectrolyte materials and the derivatives of natural products for environmental applications, aiming to add value to these materials and develop new contaminant removal technologies [94,95].

### 3.2. Agriculture

The high demand for natural biostimulants that enhance plant growth and yield has led researchers in recent years to seek renewable alternatives for innovations in agriculture. The application of biopolymer polyelectrolytes demonstrates high potential for use in agriculture [96,97]. It is here that the use of polymers, particularly polyelectrolytes, in agriculture has aided in increasing water storage capacities and efficient water use [98].

#### 3.2.1. Soil Erosion Control

Good soil is essential for high agricultural productivity, making soil erosion a significant problem that threatens agricultural productivity and, consequently, food production. The effects of erosion include nutrient loss, decreased fertility, desertification, and the denudation of the topsoil layer [99,100].

To improve the hydrophysical properties of soil, prevent erosion, and act as soil conditioners, research has been conducted on polymeric interpolyelectrolyte complexes (IPECs). IPECs can stabilize the topsoil layer by using water-soluble natural or synthetic polymers, which bind to soil particles and form a protective layer (crust) on the soil surface [101,102,103,104]. Studies found that the effectiveness of IPECs in stabilizing sand and soil against wind erosion and water retention depends on their chemical composition and soil nature (e.g., sod-podzolic soil and sand). IPECs in which only a small proportion of polycationic units were neutralized by polyanionic units proved to be the most effective, presenting more stable crusts due to electrostatic and hydrophobic contacts. Panova et al. demonstrated that soils treated with IPECs exhibited impressive mechanical resistance, withstanding pressures of up to 40 MPa, and remarkable wind resistance, enduring speeds of up to 190 km/h [105]. Orazzhanova studied the IPEC complex of chitosan and polyacrylic acid in a 1:9 ratio, observing a significant improvement in soil structure and resistance to deflation. Soil structural elements also showed 99% resistance to water erosion. Additionally, Klivenko et al. used biodegradable polymer IPECs and tested soil structures with individual polymer solutions and mixtures, obtaining the following sequence in ascending order of Young’s modulus: gellan gum (GG) < chitosan (Ch) < sodium alginate (SA) < sodium carboxymethylcellulose (SC) < Ch-GG < Ch-SA < Ch-SC. For Ch-SC, the maximum resistance value was 430 kPa [106,107]. Furthermore, Lerma et al. developed a hybrid compound utilizing montmorillonite and poly(acrylic acid) mimicking the structural and functional properties of humified soil for soil retransformation strategies. By modifying montmorillonite with N-vinylbenzyl-N-trimethylammonium chloride monomers and crosslinking with acrylic acid, the researchers achieved materials that closely resembled soil humus, offering the potential for degraded soil rehabilitation [8].

#### 3.2.2. Improved Crop Yields and Nutrient Management

Among the essential nutrients plants require for efficient crop production, nitrogen stands out as the most crucial in nearly all metabolic activities. This makes it the critical nutritional factor for optimal crop yield and development. However, excessive nitrogen application has negatively impacted the environment, leading to surface water eutrophication and soil and water resource acidification, which, in turn, is detrimental to producers and consumers. To control soil nitrogen levels, various strategies have been adopted to enhance its efficiency [108,109]. One strategy to counteract excessive fertilizer loss to the environment is to use PECs as polymeric matrices controlling the release of fertilizers (nitrogen) and nutrients (phosphorus, zinc, and potassium, among others).

For instance, Rychter et al. evaluated the capacity of poly(methylene-co-cyanoguanidine) (PMCG) as a nitrogen fertilizer against two plant types: monocotyledonous oats (Avena sativa) and dicotyledonous radish (*Raphanus sativus* L.), which were incubated for 6 months [9]. They determined that PMCG released nitrogen-based degradation products, promoting the growth and development of the green parts of both plants. PMCG achieved these results because it is an oligomeric polycation with a high content of nitrogenous compounds. In another study, Salachna et al. used gellan gum to evaluate the effects of depolymerization on the growth and antimicrobial activity of two ornamental species, *Eucomis bicolor* and *Eucomis comosa*, used in natural medicine. The biopolymer was applied using polyelectrolyte complexes, and the results compared with control samples showed increased biomass, greater flowering, and increased potassium content in the leaves [110]. Li et al. and their collaborators used the layer-by-layer electrostatic self-assembly (LBL) technology to prepare an environmentally sensitive (pH) fertilizer with layers of natural polyelectrolytes chitosan and lignosulfonate deposited on zinc phosphate and ammonium coated with polydopamine (Pdop). They determined that the nutrients N, P, and Zn had different release profiles, specifically, the release rate is faster in an alkaline pH, associated with the disassembly and degradation of natural polyelectrolytes. Additionally, they found that the obtained fertilizer improves nutrient utilization efficiency, promoting plant growth when used in maize plants (in pots) for 20 days [111]. Feng et al. developed a controlled/slow-release fertilizer (CSRF) consisting of layers of polydopamine deposited on a surface of zinc phosphate and ammonium, which supply nutrients to be released in a controlled manner. To achieve controlled release, poly (N,N-dimethylaminoethyl methacrylate) (PDMAEMA) chains were immobilized on the layers using the surface-initiated atom transfer radical polymerization method. The LCST, or Lower Critical Solution Temperature, is a critical point in the phase transition of certain polymers, such as the PDMAEMA mentioned here. In this context, the LCST of the PDMAEMA directly affects the nutrient release rate depending on temperature and pH changes. Below the LCST in a basic medium, the nutrient release rate may accelerate, whereas above the LCST in the same medium, the release rate may decrease. This understanding is crucial for designing effective and controllable nutrient release systems in tissue engineering applications [112].

Meurer et al. fabricated a foliar fertilizer based on pH-sensitive biohybrid microgels. Initially, they synthesized the microgels using the inverse mini-emulsion polymerization method (water in oil, W/O), which involved crosslinking poly(allylamine hydrochloride) (PAH) and N,N′-methylene-bis(acrylamide) (BIS) through an Aza-Michael addition mechanism. Subsequently, the microgels were grafted with peptide fusion protein to promote foliar adhesion. The results presented indicate that the microgels can be loaded with different amounts of micronutrients (Fe^3+^), and the presence of proteins allows for strong binding to leaf surfaces. They also analyzed the ability of the biohybrid microgels to counteract iron deficiency in cucumber leaves, demonstrating a “greening” effect after 48 h, indicating an increase in chlorophyll content [113]. Hui-li et al. developed a double-layer slow-release fertilizer (SRF) for nutrient release and improved water retention. The SRF consisted of coating urea with an inner layer of polyvinyl alcohol (PVA) and methylcellulose (MC), while the outer layer incorporated attapulgite in polyacrylic acid (PAA) and natural biodegradable lignin grafted with polyacrylamide (PAM). The double-layer slow-release fertilizer (PML-SRF) achieved release equilibrium at 42 days, with an accumulated urea release rate of 85.22%. Moreover, soil water retention capacity reached 62.46% when adding 4 g of PML-SRF to 200 g of dry soil [114]. 

**Figure 8:**
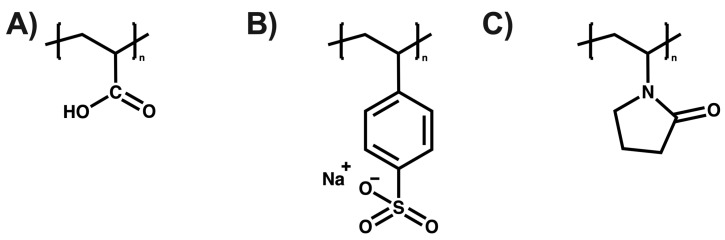
(**A**) poly (acrylic acid), (**B**) poly (sodium styrenesulfonate), and (**C**) poly (vinylpyrrolidone).

Elhassani et al. utilized a chitosan (CS), polyvinyl alcohol (PVA) (50:50), and lignin nanoparticle (LNP) coating to encapsulate urea granules. The formulation with 3% LNP exhibited superior physicochemical properties. This CS/PVA-LNPs (3%) formulation demonstrated good nitrogen slow-release properties over time when applied at a 7% coating level, achieving an 18-day release compared to the uncoated urea released within 24 h [115]. Zhang et al. formulated a double-layer SRF to coat urea. The SRF comprised an inner layer of lignin–bentonite clay crosslinked with alginate and an outer layer of poly (acrylic acid) (PAA), which enhances water adsorption capacity. The SRF-PLC showed a urea release rate in soil of 43% and in water of 74% over 5 days, slower than the control sample (83% in soil and 87% in water). Furthermore, water absorption improved by 32%, attributed to the outer layer (PAA) enhancing water retention [116]. Finally, Mercado et al. evaluated the effects of poly (acrylic acid), poly (sodium styrene sulfonate), and poly(vinylpyrrolidone) (See Figure 8) on the aqueous phase of acidic sulfate soils in the Cordoba department of Colombia to understand the chemical properties of these soils. The results indicated that the chemical species present in the soil’s aqueous phase and the functional groups of the polyelectrolyte contribute to the immobilization of the present ions [117].

#### 3.2.3. Biodegradation and Ecotoxicity

In the realm of modern agriculture, two critical challenges are bioaccumulation in the food chain and ecosystem contamination, leading to the urgent need to develop sustainable agricultural practices that minimize the release of toxic compounds into the environment. In this context, polyelectrolytes have emerged as a technology to efficiently deliver agrochemicals or biological compounds. Garces et al. advanced in this direction by developing artificial microorganism growth systems based on biodegradable hydrogels for the use of plant growth-promoting inoculants, using citric acid, sorbitol, dextrose, starch, and agar as polymer precursors. The results obtained show that the polymeric matrices are suitable growth media for plant growth-promoting microorganisms [118]. Following this line of research, Zhao also utilized the layer-by-layer (LBL) technique to encapsulate the poorly water-soluble herbicide picloram (PLR) using chitosan (CS) and sodium lignosulfonate (SL). The results showed that the photodegradation rate of PLR in microcapsules decreased with an increasing number of layers. The same trend was observed in the mean release rate (t50), where the microcapsule with 12 layers of polyelectrolytes loaded with PLR reached values of 2.26 h, compared to the microcapsule with 4 layers, which showed a t50 of 0.43 h [119]. On the other hand, the encapsulation of tetrahydrocurcumin (THC), a natural fungicide, in OSA-starch or chitosan matrices presented promising results. Chitosan particles loaded with THC demonstrated superior antifungal properties, especially effective against Fusarium graminearum, a pathogen causing severe diseases in crops such as wheat, corn, and barley. These advances not only improve the effectiveness of natural fungicides but also reduce the accumulation of harmful mycotoxins in crops [120].

Through these studies, it is clear how innovation in the biodegradation and controlled delivery of agrochemicals can significantly contribute to sustainable agriculture, simultaneously addressing the challenges of ecotoxicity and bioaccumulation. These advances not only enhance the safety and efficacy of agricultural treatments but also pave the way for more environmentally friendly agricultural practices.

#### 3.2.4. Emerging Technologies and Innovations for Agriculture through PELs

One of the most significant promises for modern agriculture is the utilization of plant growth-promoting bacteria (PGPB) and plant growth-promoting fungi (PGPF), which play a crucial role in soil restoration and increasing crop productivity. This approach underscores the importance of developing advanced techniques to protect these microorganisms from adverse environmental factors and ensures their controlled release into the environment. In this context, Mohammadinejad et al. explored the encapsulation of Pseudomonas fluorescens and Bacillus subtilis using a combination of sodium alginate, gelatin, carbon nanotubes, and SiO_2_ nanoparticles. The resulting nanocapsules not only enhanced bacterial proliferation but also significantly increased the root length of the treated plants, thus demonstrating the potential of this technology to improve plant growth [121,122].

Advancements in encapsulation technology offer innovative solutions for enhancing the efficacy of beneficial microorganisms in agricultural practices. For example, Kadmini et al. demonstrated how the encapsulation of *Pseudomonas fluorescens* and *Azospirillum brasilense* strains can be achieved using halloysite and alginate [Ha-Ag] or montmorillonite and alginate [Mt-Ag]. The study revealed that Ha-Ag capsules maintained bacterial survival, with bacterial counts reaching 14.8 log CFU g^−1^ after 3 months. Additionally, the swelling and biodegradability properties were also analyzed, with the montmorillonite and halloysite formulation reaching between 61.5 ± 1.35% and 36.5 ± 5% swelling ratio, respectively. The biodegradation rate was higher for the A. brasilense strain encapsulated with Ha-Ag, reaching approximately 40% in 30 days. The application of Ha-Ag capsules for the *A. brasilense* strain to wheat plants increased root and shoot biomass by 100%, and the height was 25% higher than the control plants. This same formulation provided higher amounts of N and P in wheat plant shoots and roots [123].

In another study, Mancera-López et al. investigated the encapsulation of *Trichoderma harzianum* using different methods: (a) drip encapsulation and (b) internal emulsion/gelation. The results showed that medium-sized capsules (1.5 ± 0.3 mm) obtained by the drip method favored the production of released conidia (1.5 × 108 conidia per mL) in submerged culture and better stability against UV radiation. The antagonistic activity of T. harzianum against different phytopathogenic fungi showed no significant differences regarding the methodology used or the size of the generated capsules. Finally, they observed a cell viability loss of approximately 30% after two years of storage at room temperature [124].

Similarly, Panichikkal et al. encapsulated *Bacillus licheniformis* in alginate with chitosan nanoparticles (CNPs) supplemented with rice starch (RS), achieving an encapsulation efficiency of 99%. The study demonstrated that encapsulation did not compromise the ability of B. licheniformis to produce beneficial metabolites such as indole-3-acetic acid (IAA) and 1-aminocyclopropane-1-carboxylic acid (ACC) deaminase, nitrogen fixation, or its antifungal properties. Furthermore, the seedlings of *Capsicum annuum* (L.) treated with encapsulated B. licheniformis exhibited improved growth, reaching a shoot length of 7.14 cm, and demonstrated the suppression of the soil pathogen *Sclerotium rolfsii* [125].

Peil et al., 2020 employed lignin-based polyelectrolyte layers (Kraft lignin and cationic lignin) to encapsulate *Trichoderma spores* using the layer-by-layer (LBL) technique. Their findings indicated that encapsulation did not affect spore viability or germination control. Moreover, an in vitro study revealed that the enzymatic degradation of the lignin layers triggered the germination of encapsulated Trichoderma spores in response to an esca pathogen attack, thereby unleashing their antagonistic potential against fungal pathogens [126].

### 3.3. Medicine

**Figure 9:**
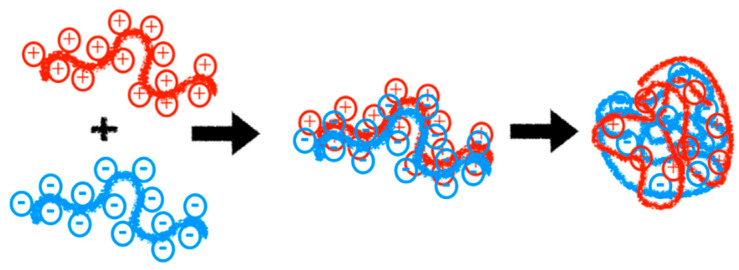
Formation of polyelectrolyte complexes by the interaction of polymers with opposite charges.

Polyelectrolytes have been utilized as matrices for oral drug delivery, nasal, and buccal routes due to their bioadhesive properties, for tissue regeneration therapy, the surface modification and functionalization of implants, the construction of artificial organs, gene therapy, and anticancer treatment [15,127,128,129,130]. In the last decade, the formation of polymeric complexes, also known as polyelectrolyte complexes, has been employed for the controlled and injectable delivery of pharmaceuticals to specific sites [15]. The formation of polyelectrolyte complexes occurs through the interaction of polymers with opposite charges, leading to the formation of a complex system. These formations are driven by strong electrostatic attractions without the need for reaction initiators, catalysts, or crosslinkers (See Figure 9) [131,132]. Below, we discuss various studies highlighting the use of polyelectrolytes in medicine for applications such as drug delivery, genetic material transportation, and the administration of proteins, peptides, and more.

#### 3.3.1. Drug Administration

Drug delivery is one of the strategies where polyelectrolytes play a fundamental role in complementing the limitations of conventional drug delivery systems such as oral or injectable methods, in addition to achieving control over release times and potential side effects [133]. Sarmento et al. prepared polyelectrolyte complexes from chitosan and dextran sulfate with insulin incorporation, achieving association efficiencies of 50% and 97% according to acquisition parameters such as charge ratio, pH, and insulin theoretical charge [127,134]. Zhang et al. used chitosan and carboxymethylated starch for lysosome encapsulation for delivery in intestinal systems, demonstrating them to be good carriers for targeted delivery [135]; Cerchiara et al. formed chitosan and carboxymethyl cellulose microparticles for vancomycin release to combat the antibacterial activity of Staphylococcus aureus in the stomach, showing promising results for drug administration in the colon [136]. Bigucci et al. obtained the complexes of chitosan and carboxymethylcellulose for local chlorhexidine delivery as vaginal inserts against antimicrobial activity, finding that the polyelectrolyte complexes exhibited antimicrobial activity against pathogens responsible for vaginal infections [137]. The other examples of polyelectrolyte application in drug release are shown in Table 2.

Recent studies and innovations in drug delivery have improved efficiency, administration rate, time, and the location of release in different therapeutic processes, achieving the preservation and maintenance of adequate drug levels at target sites by controlling parameters such as toxicity, release profile, and biodegradability [147]. Polyelectrolytes have been materials that, due to their physicochemical characteristics, present a great selectivity for drugs and the possibility of controlling release without side effects or premature excretion, so different alternative approaches have been studied for oral, buccal, rectal, intravenous, intramuscular, or subcutaneous drug administration [148], such as obtaining multilayer polyelectrolyte films, nanoparticles, hydrogels, or other types of materials from PELs.

##### Polyelectrolyte Multilayers (PEMs)

**Figure 10:**
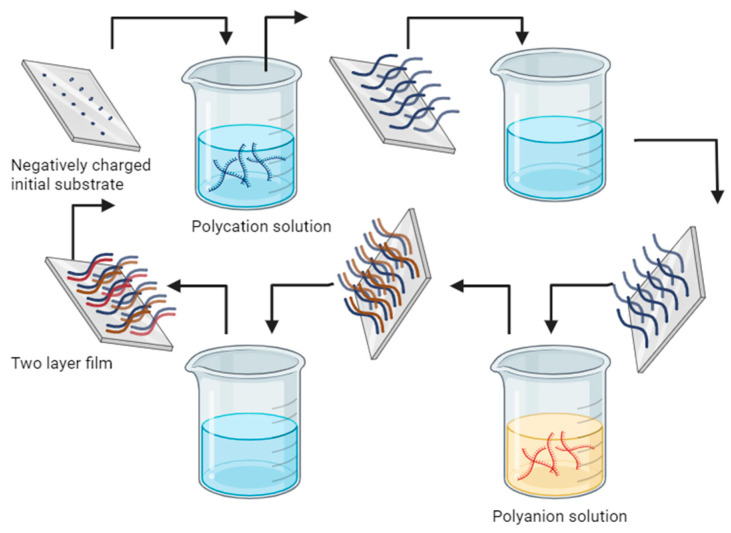
Schematic of the substrate adsorption and double layer film formation in polycation and polyanion solution [151].

Polyelectrolyte multilayers (PEMs) are nanostructured systems formed by the sequential deposition of electrically charged polymers on a solid surface or colloidal core. These systems are based on the electrostatic interaction between the charged groups of the polymers, allowing the formation of multiple alternate layers [149], although in recent years, different interactions have been studied for obtaining materials such as covalent bonds, hydrogen bonds, Van der Waals forces, or the hydrophobic interactions of polyelectrolytes [150]. The layer-by-layer (LBL) method (See Figure 10) is an efficient, easy, and economical alternative for the self-assembly fabrication of ultrathin films; it is based on the adsorption of polyanions and polycations for the formation of ultrathin polyelectrolyte multilayers; it involves the alternate immersion of the substrate or core in solutions containing polyelectrolytes of opposite charge, so that each adsorbed layer adheres to the surface of the previous substrate through electrostatic interactions and can be controlled in terms of thickness and composition [151,152,153]. These materials have proven to be colloidal supports for drug loading and release, with the possibility of controlling release kinetics based on LBL encapsulation, improving physicochemical properties, permeability, half-life increase, active compound protection, and material stability enhancement [150,154].

A variety of materials have been used in sustained-release systems based on this technique, some examples of commonly used materials include natural polyelectrolytes due to their biocompatible and biodegradable characteristics, making them ideal for use in medical treatments. For example, the material developed by Onnaity, R. et al., based on a thin film of independent polyelectrolytes from biopolymers such as 85% deacetylation degree chitosan and low viscosity sodium alginate (39% G or guluronic acid), to predict drug interactions with mucous surfaces of the intestinal, respiratory, genital, and ocular tracts, protect body organs in contact with the environment by recreating mucous mimetic matrices to provide information on the biophysical interactions between the body’s protective barrier (mucosa) against different drugs or external aggressive agents, optimizing the administration of different drugs for different diseases, such as cystic fibrosis or cancer [155].

On the other hand, multilayer systems have also been used for the controlled release of antibiotics and to fight bacterial infections as in the case of Cai, H. et al., who developed multilayer films through electrostatic self-assembly, antibacterial and anti-adhesive with polyelectrolyte nanoparticles for the transport of hydrophobic biocides using biopolymers such as tannin and chitosan embedded with Triclosan micelles, obtaining a material with excellent pH-sensitive release activity in acidic environments with an efficiency of 99.62% and 97.35% after 24 h of incubation at pH 6 against E. coli and P. aeruginosa, respectively, with an antimicrobial capacity of 30 days [156]. On the other hand, cytotoxic drugs such as doxorubicin have been encapsulated in multilayers for localized tumor treatment and reduction in side effects. Moreover, Daubiné, F. et al. reported obtaining a material with multilayers of polyelectrolytes that has been used for the prevention of in situ bone metastasis, as reported in their study by obtaining multilayer films of covalently grafted polylysine with β-cyclodextrin and charged with the antitumoral Biphosphonate risedronate, a material with biological properties for controlled cell activation or local drug administration, which also improved with complexation the inhibition of cancer *cells* “in vitro” [157]. M-Y et al. reported obtaining a multilayer film of nano-molded block copolymer modified from polyethylene imine and an electroactive derivative of poly(3,4-ethylenedioxythiophene), which has shown different advantages such as a large amount of drug load, resulting in high efficiency in drug administration, in addition to a greater retention of the bioactive molecule, compared to materials used for sustained drug release to cells suspended in a solution with constant concentrations over time of released molecules, due to the incorporation of nano structural characteristics in the material providing a greater control of space–time in cellular functions and allowing the release of bioactive molecules after the application of an external electrical stimulus, a technology that is also being studied for possible application in therapeutic implants and tissue engineering [158].

These are just a few examples of materials and therapeutic applications in which sustained-release systems based on polyelectrolyte multilayers have been studied and applied. The wide variety of material options and design possibilities offer significant potential for the development of more effective and personalized therapies in the field of medicine.

##### PEL Hydrogels

Since their discovery in 1954, hydrogels have been widely used in medical applications due to characteristics such as biocompatibility, low toxicity, and biodegradability. However, being three-dimensional polymeric networks of electrically charged and crosslinked polymers, and highly hydrophilic, their application in drug transport has been limited as they release drugs rapidly. Therefore, a subject of study is the control of material diffusivity using modification techniques to control different material properties [147,159].

Polyelectrolyte hydrogels are considered a viable option for controlled drug release due to their unique properties, providing several advantages such as allowing sustained and controlled release of encapsulated drugs and maintaining a constant therapeutic concentration at the site of action. This property is beneficial for long-term treatments or chronic diseases since maintaining a constant and controlled concentration of the drug at the site of action yields more effective and consistent results [160]. Polyelectrolyte hydrogels are an attractive option for controlled drug release due to their advantages in therapeutic efficacy, reduced side effects, and treatment customization capability. This technology is the subject of intense research and offers great potential in the development of safer and more effective therapies [161,162]. Zhang et al. reported the development of a hydrogel from carboxymethyl chitosan, a highly soluble derivative of chitosan, with good biocompatibility and hydration properties for various medical applications. This was prepared by crosslinking due to the introduction of Schiff bases and coordination bonds with hyaluronic acid designed for drug transport, resulting in a material with excellent swelling, biodegradability, and antibacterial properties, achieving the release times of acetylsalicylic acid of over 120 h [163].

**Figure 11:**
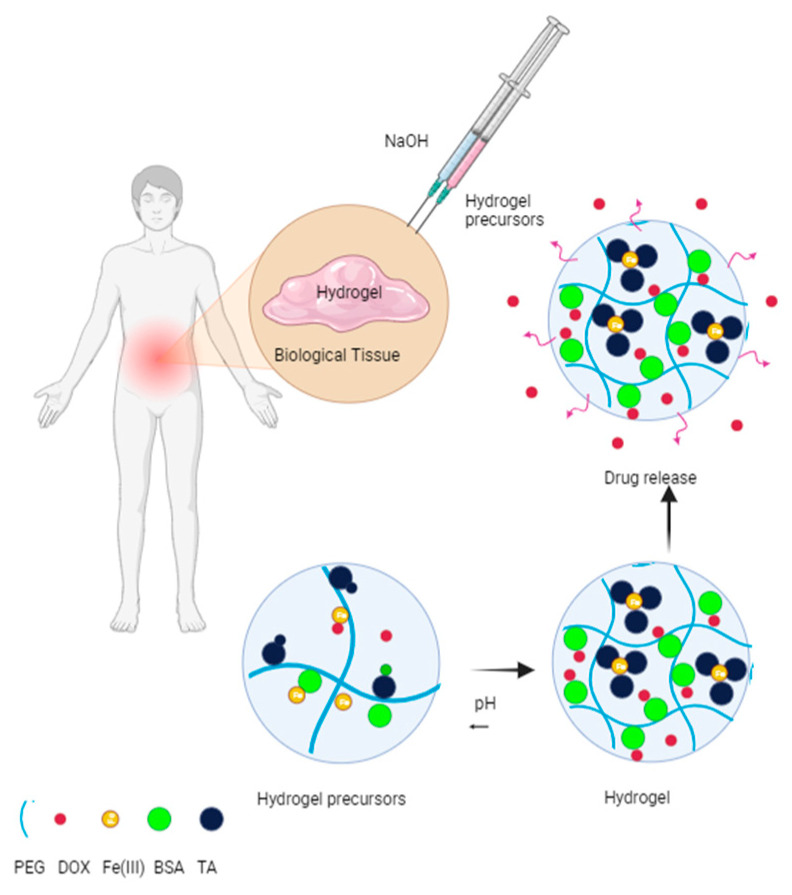
Injectable hydrogel for personalized cancer treatment [164].

Similarly, the possibility of establishing personalized treatments that allow adjusting the speed and profile of drug release according to the specific needs of each patient and disease, offering greater flexibility to adapt the treatment, has led to the development of materials such as crosslinked polyethylene glycol hydrogels with albumin and Metal–Phenolic complex (See Figure 11). Bubpamala et al. reported the development of polyethylene glycol hydrogels crosslinked with albumin and Metal–Phenolic complex. They reported that this pH-sensitive hydrogel, with supramolecular crosslinked bonds, is advantageous because it provides invasive access to internal tissues, lasting up to three weeks in the organism. Loaded with doxorubicin, this material also exhibits versatile mechanical, rheological, and morphological properties depending on the concentrations of hydrogel components, influencing drug release times and “in vitro” biocompatibility [164].

It is important to note that research in this field is still evolving, and efforts are being made to improve the release efficiency, stability, and biocompatibility of polyelectrolyte hydrogels for eventual clinical application. The protection of encapsulated light-sensitive drugs and storage times are some of the new study objectives for material acquisition [165,166]. Authors report the obtaining of smart hydrogels whose mechanical and thermal properties vary in different environments and in response to different stimuli, as they change their conformation in aqueous solutions, allowing them to adapt and thus expand their use in the controlled release of various drugs [167]. This adaptation of the different material properties has been controlled using methods such as chemical modification, as in the hydrogel obtained by Ding et al., where selective protection and deprotection processes of thiol-ene click amino groups of chitosan were performed, obtaining an intelligent chitosan hydrogel crosslinking UV in vivo with more regular and homogeneous networks with fewer radicals formed, less toxicity, and much higher yields, for cumulative release opposing the different pHs of doxorubicin in 4 h of up to 96% and bovine serum albumin of 53% at pH 6.8 [168].

##### PEL Nanoparticles

Polyelectrolyte nanoparticles are another interesting strategy for controlled drug release as advances in nanotechnology, particularly the development of nanoparticles, have had a significant impact on regenerative medicine and healthcare by improving drug solubility, modulating release characteristics, directing molecules to desired sites, and modulating release kinetics according to treatment needs and pharmacokinetic requirements in combination therapies and targeted tissue administration [169]. However, technical and biological challenges persist in nanoparticle stability in biological environments, responsiveness to specific stimuli, and the ability to scale production to clinical levels, remaining areas of research and development.

Obtaining nanoparticles based on natural polyelectrolytes such as chitosan is attributed to their efficiency in drug transport processes, as their size easily penetrates the cell membrane and reaches the bloodstream. Rahman and Khalil reported obtaining chitosan/PEG nanoparticles for the controlled release of drugs such as isatin, an analgesic, anti-HIV, anti-inflammatory, antitumor, and anticariogenic drug, among others. The encapsulation of the drug in nanoparticles resulted in a complex formation, suggesting an improvement in therapeutic effects after treatment [170]. Similarly, bioinspired nanoparticles based on polydopamine for drug release on the skin were reported by Chen et al. They presented porous and non-porous mesostructures, which, after different tests, concluded that they would reduce the photodegradation of retinoic acid as well as increase drug loading capacity [171]. Likewise, nanoparticles used for cancer therapy were reported by Escareño et al. using microfluidics-assisted conjugation in laminar flow to establish a directed and reproducible assembly. They conducted “in vitro” doxorubicin release tests, showing a release between 45 and 65% after 24 h [172,173]. Researchers have also reported the release of doxorubicin, for hepatoma treatment, using different Shiff base reactions for drug self-assembly in nanoparticles, achieving in vivo up to 70% release at 103 h [174,175].

#### 3.3.2. Tissue Engineering

The application of polyelectrolytes in tissue engineering is an innovative strategy leveraging their electrostatic properties and versatility to promote tissue regeneration and functionality. Polyelectrolytes have become fundamental building blocks in tissue engineering due to their interaction with cells and biological molecules, as well as their ability to modulate biomolecule release through creative strategies, leading to notable advances in various medical fields [176,177].

**Figure 12:**
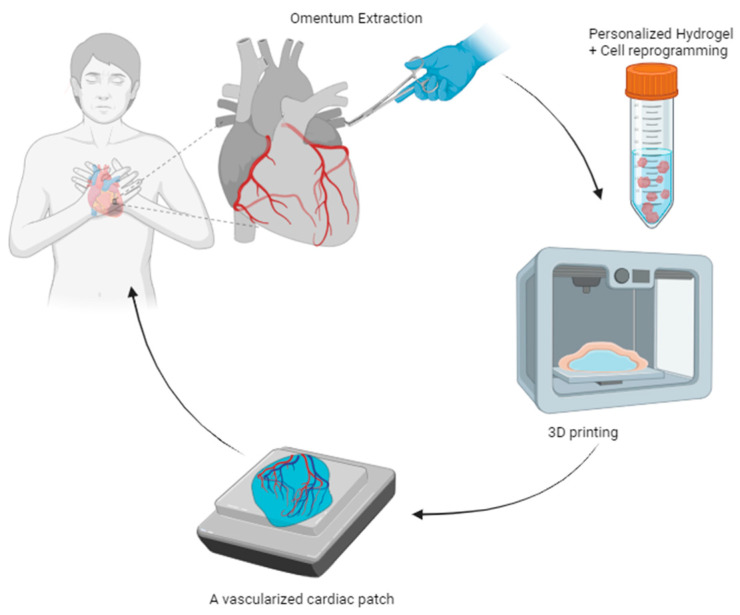
Conceptual scheme of 3D printing of customized thick and perfusable cardiac patches [182].

Polyelectrolyte hydrogels play a prominent role in tissue engineering, combining the versatility of polyelectrolytes with the water retention properties and three-dimensional structure of hydrogels. These systems form electrically charged polymeric networks in water, resulting in materials mimicking biological tissues with modulated surface charge and flexibility in incorporating bioactive molecules [178,179]. In recent years, polyelectrolyte hydrogels have found applications in advanced tissue manufacturing techniques, such as customized 3D bio-printing. This technique allows the deposition of successive layers of polyelectrolyte hydrogels, enabling the creation of scaffolds and structures with microscopic precision. The choice of polyelectrolytes and printing conditions influences the mechanical and chemical properties of the resulting material, which can be tailored to the patient’s anatomy using computed tomography [180,181]. Noor et al. report a customized vascularized hydrogel patch in the form of sodium alginate and cardiac cells, matching the atomic structure and biochemical components of each individual, a material that does not provoke an immune response after transplantation. Thus, complex biological structures have been developed with a natural architecture with great potential for the replacement of organs with chronic problems; after solving some deficiencies such as a greater expansion of cells required for organ designs, and greater precision in printing blood vessels in terms of the interior diameters of different thick structures, the resulting autologous engineering tissue can be transplanted back into the patient to repair or replace injured/sick organs with a low rejection risk (See Figure 12) [182].

However, research continues to focus on overcoming obstacles such as optimizing polyelectrolyte–cell interaction, the long-term stability of materials, the ability to create fully functional tissues, and exploring new ways to use polyelectrolytes in tissue engineering [183]. This has resulted in significant advances in regenerative medicine and the creation of artificial tissues, contributing to overcoming challenges in this field, such as the generation of support matrices and scaffolds, growth factor release, and vascular tissue engineering, among others [180,184]. This shows the ongoing commitment to perfecting the integration of polyelectrolytes in tissue engineering, which ultimately could lead to more effective and personalized solutions for tissue regeneration and the restoration of damaged tissues.

The creation of support matrices and three-dimensional scaffolds for tissue engineering is essential to facilitate cell adhesion, proliferation, and differentiation. These matrices offer a conducive environment for cell growth and tissue formation, as the charged polyelectrolytes interact with cells and can be modified to present specific functional groups, regulating cell adhesion and intercellular communication. These materials can be polymers, hydrogels, and ceramics, among others, designed with specific mechanical and chemical properties to suit the needs of the tissue to be regenerated [185,186]. In this sense, the creation of porous 3D scaffolds focuses on allowing cell adhesion, migration, and proliferation while the material degrades. Examples such as the scaffolds of Fu et al., where degradable compounds such as chitosan and collagen were employed, show their viability by allowing cardiac cells to adhere, differentiate, and maintain their biological activity [187]. Another approach is that of Heydari and Esmaeili, who have developed a polyurethane–chitosan–protein–gelatin scaffold using electrospinning, resulting in high tensile strength fibers with anticoagulant properties and high cell viability, making it suitable for cardiac applications [188]. Unagolla et al. explored the microparticles of the polyelectrolyte complex of chitosan and carboxymethylcellulose, where they have reported better mechanical properties both in dry and wet states, as well as an increase in cell proliferation in “in vitro” culture studies. This positive effect is attributed to the addition of 20% calcium phosphate in the material structure. These findings illustrate how polyelectrolyte properties can be optimized and improved to further drive advances in tissue engineering and cell regeneration [189]. In a similar line, Jin et al. have reported another relevant study focused on the “in vitro” cultivation of chondrocytes in polyelectrolyte complex hydrogels such as native chitosan and glycolic acid. This material presents an artificial extracellular matrix with important characteristics for applications in cartilaginous tissue engineering, such as water absorption, enzymatic degradation rate, and the adjustable mechanical properties of the material [190]. Wang et al. have presented a simple and effective strategy for the formation of three-dimensional hierarchical microchannels by combining poly (diallyldimethylammonium chloride) and poly (sodium 4-styrenesulfonate). This approach has shown remarkable cell proliferation and has achieved porosity percentages of up to 76%, which could lead to increased tissue growth by up to 53% according to various “in vitro” and in vivo assays [191]. Similarly, the combination of xanthan gum and chitosan in the presence of iron oxide nanoparticles, as reported by Rao et al., has allowed the creation of magnetically sensitive hydrogels using in situ ionic complexation techniques. These hydrogels possess a well-defined porous structure, in addition to demonstrating notable cellular proliferation and adhesion, along with improvements in their mechanical properties [192]. The other examples of the application of polyelectrolytes in obtaining scaffolds and support matrices for tissue engineering are shown in Table 3.

**Figure 13:**
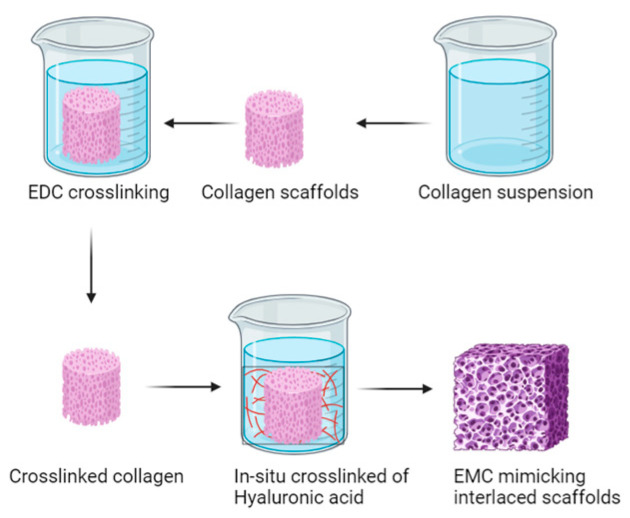
Hybrid scaffolds for cardiac valve tissue engineering based on type I collagen and hyaluronic acid [204].

In the realm of hydrogel development, Luo et al. ventured to combine alginate and gelatin to create a 3D-printed hydrogel with interconnected micropores and microchannels, aiming to explore its potential for culturing human umbilical cells. The resulting hydrogel exhibited enhanced mechanical properties, structural integrity, and favorable cellular adhesion. In vivo results demonstrated outstanding performance in facilitating vessel formation both at the material’s interface and its core, along with notable wound-healing properties [202]. On a different note, Shin et al. reported on the utilization of poly(lactide-co-glycolide) nanofiber meshes functionalized with RGD peptide (arginine-glycine-aspartate) and graphene oxide (GO). These meshes were fabricated via electrospinning, displaying crucial thermal and mechanical properties such as an increased surface hydrophilicity through copolymerization, stability under cell culture conditions, and high proliferation rates in smooth muscle cells [203]. Additionally, Nazir, R. et al. employed collagen and hyaluronic acid-based materials, utilizing carbodiimide-based crosslinking techniques to fabricate hybrid scaffolds mimicking the extracellular matrix structure (see Figure 13). Trials focusing on aortic valve synthesis revealed scaffolds with high density and varied pore sizes, showcasing their potential applicability in vascular tissue regeneration by mitigating degradation within the vascular culture medium [204].

Saurer, E. et al. introduced an innovative approach for DNA delivery to vascular tissues using stainless steel stents coated with degradable polyelectrolytes such as polyester (β-amino), resulting in uniform transference to the subendothelial tissue. This, combined with the versatile nature of the utilized layer-by-layer assembly, presents a highly suitable option for gene transfer studies in vascular tissue engineering and genetics [205]. Additionally, Terry et al. previously reported on bioactive coatings for improving stent biocompatibility using polyelectrolytes, emphasizing their potential in vascular and endovascular tissues [206].

#### 3.3.3. Wound Healing with PELs

**Figure 14:**
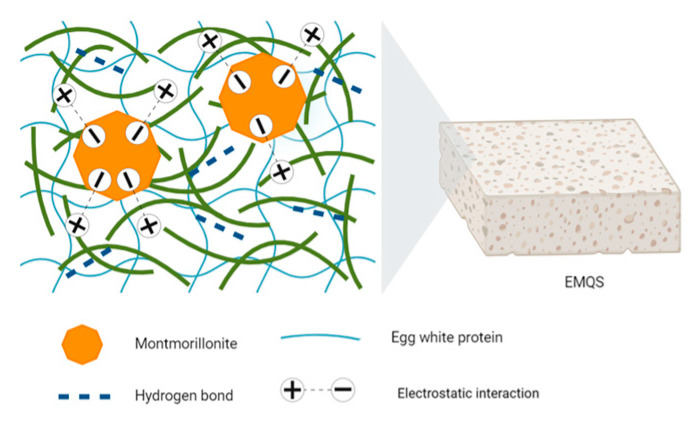
Conceptual scheme of the composite sponge structure derived from quaternized chitin, egg white protein, and montmorillonite [211].

One of the most pertinent applications of polyelectrolytes in wound healing lies in their ability to serve as carriers for the controlled administration of growth factors and bioactive molecules. This capability is particularly crucial given the clinical challenges associated with wound management, considering the significant number of individuals experiencing various types of injuries and wounds annually [207]. Li et al. developed functional dressings for severe hemorrhagic wounds with antimicrobial properties. These dressings, crafted from quaternized chitosan cotton modified with gelatin and catechol on a nonwoven cotton surface, not only promoted the healing of infected wounds but also provided the essential moist environment for recovery. In vivo trial results underscored the material’s versatility, showcasing its potential to expedite the healing of complex and bleeding wounds [208,209]. Feng et al. ventured into creating polyester fiber dressings coated with halloysite, enhancing material application and resistance to massive hemorrhages, particularly in hepatic tissues and epidermal vessels. These dressings improved hemostasis by absorbing superficial water via electrostatic interactions, thereby accelerating clot formation and platelet adhesion and contributing to effective prehospital treatment [210]. Yang et al. explored the fabrication of composite sponges comprising quaternized chitin with egg white protein and montmorillonite. These sponges exhibited antibacterial and hemostatic properties while promoting wound healing. Their high water adsorption capacity and significant porosity, combined with improved mechanical properties, accelerated angiogenesis and collagen deposition in wounds [211] (See Figure 14).

In a complementary approach, Meng et al. presented a study utilizing polyelectrolytes such as chitosan, alginate, and hyaluronic acid for composite sponge creation. Through lyophilization molding and genipin crosslinking, they achieved a material exhibiting not only high biocompatibility but also improved mechanical properties. Hemolysis assays validated its ability to accelerate blood coagulation, highlighting its potential as a wound-healing material [212]. Hassan et al. adopted a polyelectrolyte synergy-based approach. By combining chitosan and hyaluronic acid with phosphatidylcholine dihydroquercetin, they created a dressing exhibiting antioxidant, anti-inflammatory, and antibacterial properties with a direct impact on the wound-healing processes. In vivo trial results supported its potential in improving wound thickness, reducing the affected area, and promoting re-epithelialization, marking a significant advancement in skin regeneration and wound healing [213].

**Figure 15:**
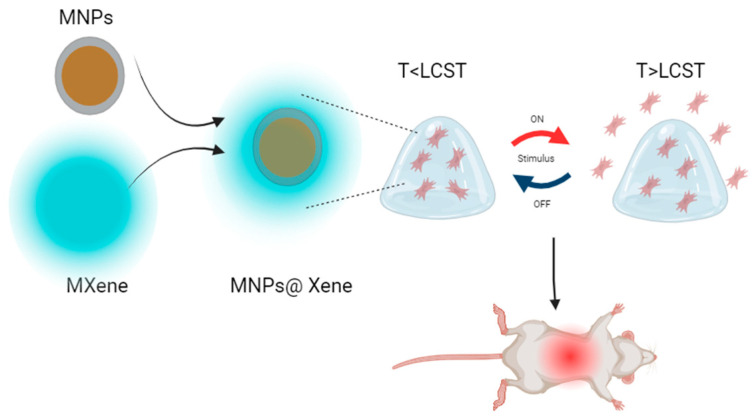
Schematic process of formation and drug release of MXene-based hydrogel system for application in infected wounds [216].

Yang et al. contributed to the field by creating a temperature-sensitive hydrogel via in situ silver ion photoreduction and acidified sol-gel transition. These polyelectrolytes based on chitosan/carboxymethyl chitosan/AgNPs resulted in a hydrogel with self-healing properties and potent antibacterial characteristics. Moreover, this material, highly biocompatible and designed to serve as a wound dressing, represents a significant step in the application of hydrogels in wound healing [214]. Wu et al. successfully obtained a gelatin hydrogel with bovine serum albumin using eco-friendly methods. This hydrogel has shown promise in treating diabetic wounds, which often pose unique challenges due to slow healing and a high susceptibility to infections. The innovative solution proposed by Wu and their team has demonstrated improved mechanical properties and immunomodulatory activity, leading to a faster and more effective healing compared to traditional approaches [215]. Additionally, Yang, X. et al. explored the use of combined polyelectrolytes, such as alginate and poly(N-isopropylacrylamide), along with MXene_3_O_4_@SiO_2_ thin-layer nanomaterials. This combination has resulted in stimuli-responsive materials capable of responding to temperature changes and administering drugs in a controlled manner. Furthermore, these materials have been shown to reduce the side effects of toxic drugs and promote wound healing, as confirmed in in vivo trials with rat models (See Figure 15) [216].

Jiang et al. have created a temperature-sensitive hydrogel that acts as a sensing interface. With the ability to connect to a Bluetooth module and emit temperature stimuli, this hydrogel has proven useful for the early diagnosis of wound infections. Comprising poly(N-isopropylacrylamide) grafted onto polyacrylic acid, vinyl-based polyacrylamide, and silver nanowires, this material acts as a temperature-sensitive interface with improved mechanical, antibacterial, and sensing properties [217]. Miguel et al. have contributed to the field with the asymmetric construction of materials through 3D electrospun printing. Their approach involves creating hydrogels composed of polyelectrolytes such as chitosan, alginate, poly(caprolactone), and silk sericin. These highly porous and mechanically improved materials have been designed to serve as skin substitutes in the healing process, offering a solution that protects against dehydration and harmful agents while promoting microbial activity, effective healing, and the regeneration of cutaneous tissue [218].

#### 3.3.4. PEL Contrast Agents

**Figure 16:**
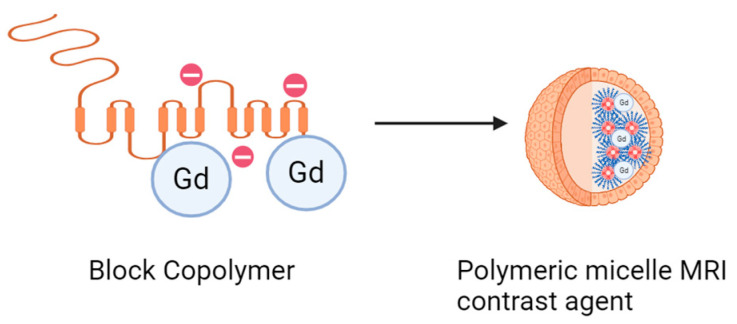
Block copolymer as contrast agent for in vivo tumor magnetic resonance micelle imaging.

Polyelectrolyte contrast agents are particles or nanoparticles that can enhance the visibility of tissues and organs in imaging modalities such as magnetic resonance imaging (MRI), computed tomography (CT), and ultrasound. The polymeric structure of polyelectrolytes allows for the encapsulation and controlled release of contrast agents, optimizing their ability to target and accumulate in specific areas of the body. This is particularly useful for early tumor detection and lesion evaluation [219]. Furthermore, the surface of polyelectrolytes can be functionalized with specific ligands that target receptors present in tumor cells or other clinically relevant sites. This targeted functionalization improves diagnostic selectivity and precision while minimizing exposure to contrast agents in undesired areas [220]. Some notable works are highlighted below: Wang, H. et al. employed a two-pronged approach involving the creation of two types of self-assembled polymer–gadolinium conjugates using methyl ether methacrylate oligo (ethylene glycol), one linear and the other crosslinked, via a reversible polymerization technique. These agents formed spherical or worm-like nanostructures and demonstrated low toxicity and hemocompatibility. The MRI signal efficacy was improved compared to small molecular agents like Gd-DTPA, especially with the linear agent in breast tumors. Although the crosslinked agent was absorbed by tumor cells, its specific degradation affected its efficacy. Both agents allowed for the clear and enduring imaging of major organs, acting as MRI angiography agents by highlighting blood vessels, even in the liver and kidney. This approach could unveil more about the relationship between the structure and function of these polymer–gadolinium conjugates (See Figure 16) [221].

Another promising strategy is the use of polyelectrolyte micelles as a vehicle to deliver MRI contrast agents specifically, as demonstrated by Shiraishi and their team. A block copolymer, PEG-b-poly(L-lysine), was employed to bind gadolinium ions via DOTA chelating residues. The DOTA halves successfully bound to the primary amine groups of lysine residues. The resulting copolymer formed polymeric micelles that maintained their structure even after partial gadolinium binding. These micelles were injected into mice and demonstrated stable blood circulation, accumulating in solid tumors due to the EPR effect. The MRI signal intensity in tumors increased significantly, improving diagnosis. This promising approach could be valuable in combination with drug-targeting systems based on polyelectrolyte micelles [222].

## 4. Conclusions

The field of polyelectrolyte research, especially in environmental, agricultural, and medical applications, has seen considerable growth recently. The inherent versatility of these materials has led to their increasing utilization in a variety of applications by researchers globally. In environmental science, there is an acute global focus on the preservation of aquatic ecosystems. Polyelectrolyte-based materials and their derivatives are pivotal in advancing technologies that mitigate the environmental impacts of anthropogenic activities and industrialization. Concurrently, sustainable agriculture is gaining emphasis with aims to satisfy the escalating food demands of the world’s population while minimizing environmental, human, and productivity impacts. In response to these goals, polyelectrolytes have demonstrated numerous beneficial properties—such as protection, controlled release, and safety—for various agricultural applications. Yet, many potential uses remain under-researched, marking polyelectrolytes as a material of great promise for eco-friendly farming. However, challenges such as scaling up production remain. Regarding medical applications, the broad spectrum of uses significantly enhances practices and technologies in this critical sector. Nonetheless, polyelectrolytes encounter hurdles in bioavailability, toxicity, controlled release, specificity, complex design, immunological responses, standardization, regulation, tailored design, and economic feasibility. The high costs associated with large-scale production and integration into effective clinical therapies necessitate a focus on the economic viability and scalability of these strategies. As research advances, overcoming these obstacles will be crucial for optimizing the potential of polyelectrolytes in healthcare and ensuring their safety and efficacy in clinical settings. In summary, the marked expansion in the realm and applications of polyelectrolytes substantially contributes to our understanding of how materials can be intricately engineered and customized for vital applications across critical sectors globally, including environmental conservation, agriculture, and healthcare.

## Figures and Tables

**Table 1:** Documents with the highest citations in worldwide research on polyelectrolytes and their applications.

Title	Journals	Number of Citations	Number of Citations per Year	References
Chitosan—A versatile semi-synthetic polymer in biomedical applications	*Process in Polymer Science*	2167	164.53	[16]
Electrophoresis of DNA and other polyelectrolytes: Physical mechanisms	*Reviews of Modern Physics*	649	27.04	[17]
Detailed Structure of Molecularly Thin Polyelectrolyte Multilayer Films on Solid Substrates as Revealed by Neutron Reflectometry	*Macromolecules*	559	21.50	[18]
Protein Encapsulation via Porous CaCO_3_ Microparticles Templating	*Biomacromolecules*	486	24.30	[19]
Smart Electronic Yarns and Wearable Fabrics for Human Biomonitoring made by Carbon Nanotube Coating with Polyelectrolytes	*Nano Letters*	474	29.62	[20]
An environmentally benign antimicrobial nanoparticle based on a silver-infused lignin core	*Nature Nanotechnology*	451	50.11	[21]
Stabilization of aqueous nanoscale zerovalent iron dispersions by anionic polyelectrolytes: adsorbed anionic polyelectrolyte layer properties and their effect on aggregation and sedimentation	*Journal of Nanoparticle Research*	449	28.06	[22]
Trends in polymer electrolytes for secondary lithium batteries	*Journal of Power Sources*	447	18.58	[23]
Design of Antibacterial Surfaces and Interfaces: Polyelectrolyte Multilayers as a Multifunctional Platform	*Macromolecules*	403	26.86	[24]

**Table 2:** Applications of polyelectrolytes as carriers of therapeutic molecules.

Polyelectrolytes	Therapeutic Molecules	Ref.
Chitosan and dextran sulfate	Recombinant human insulin	[138]
Chitosan and carbixymethylcellulose	Vancomycyn	[136]
Chitosan	Insuline	[139]
Alginate–Polyethyleneimine beads	Furosemide	[140]
Poly (diallyl dimethylammonium chloride) (PDADMAC) and polystyrene sulfonate	Daunorubicin	[141]
bis-(2-aminopropyl) poly (ethylene glycol) and poly(methacrylic acid)	Bovine serum albumin	[142]
Chitosan and Poly (γ-glutamic acid)	Stromal-derived factor-1	[143]
Poly(ethylenimine) modified by deoxycholic acid	siRNA	[144]
Poly (ethylenimine)	Plasmid DNA	[145]
Chitosan and Dextran sulfate	Curcumin	[146]

**Table 3:** Applications of polyelectrolytes as scaffolds in tissue engineering.

Polyelectrolytes	Material Type	Ref.
Chitosan and sodium alginate	Hydrogel	[193]
Chitosan and polygalacturonic acid	Thermosensitive injectable hydrogel	[194]
Poly (lactic-co-glycolic acid) and Collagen	Powder–Composite scaffold	[195]
Alginate/sericin/graphene oxide	Nanocomposite hydrogel	[196]
Chitosan and montmorillonite	Nanoparticle scaffold	[197]
Chitosan and its derivatives in 3D/4D (bio) printing	Hydrogel	[198]
Polycaprolactone–Polyethylene Glycol	Nanofibrous scaffold	[199]
Kappa-carrageenan	Sprayable and injectable visible-light Hydrogel	[200]
Methacrylated pullulan/polyethylene (glycol) diacrylate composite	Hydrogel	[201]

## Data Availability

Not applicable.
